# Supplementary figures and images for: The Pseudomonas aeruginosa ribonuclease Ribocin cleaves eukaryotic ribosomes at helix 69 to inhibit host translation
Source: PLoS Biol. 2026 May 20;24(5):e3003790. doi: 10.1371/journal.pbio.3003790 (PMC13189344; doi:10.1371/journal.pbio.3003790)

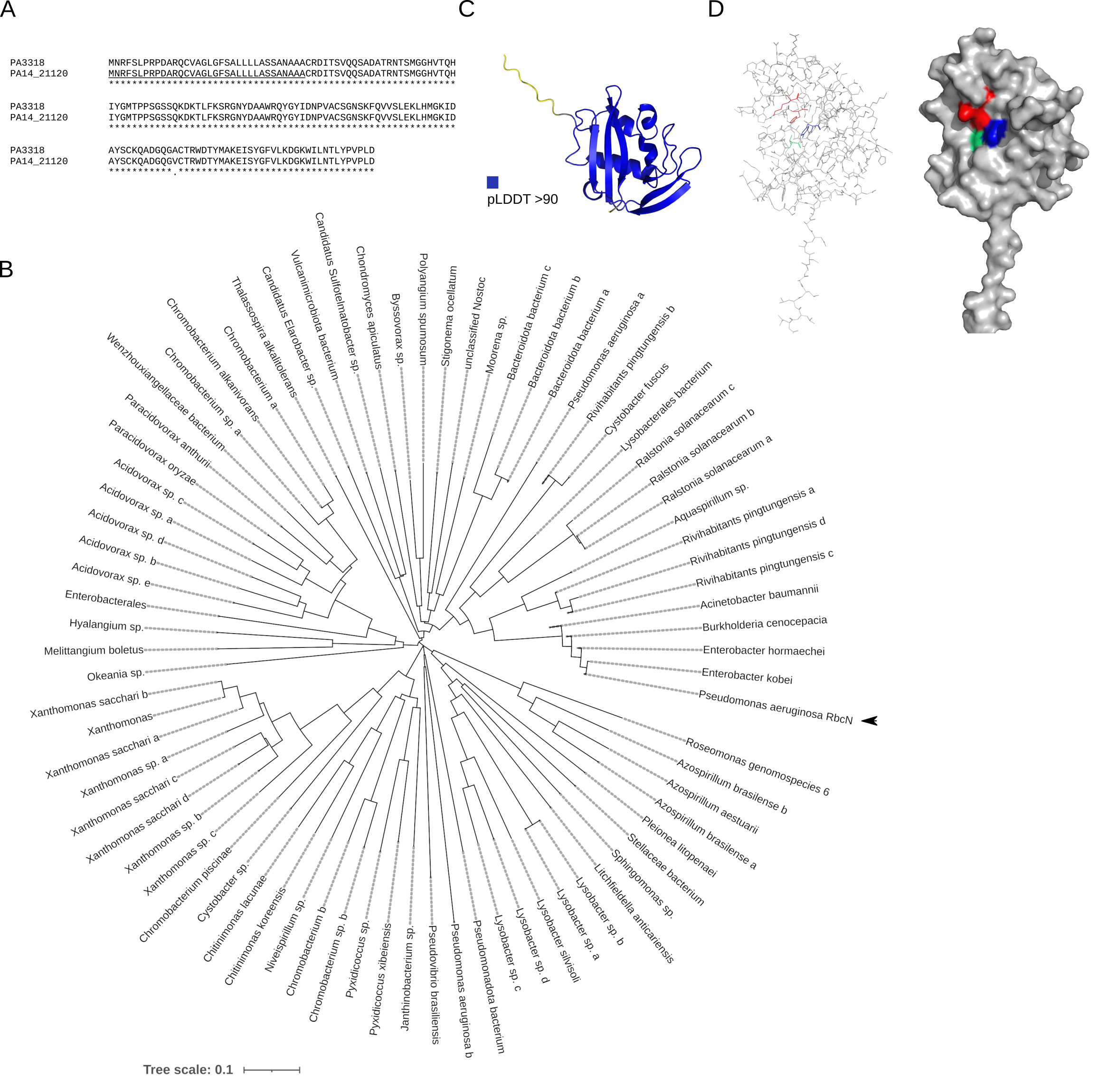

Supplement: S1 Fig — (A) Protein sequence alignment of PA14_21120 and its ortholog PA3318 in P. aeruginosa strain PA01. The signal peptide sequence is underlined. (B) Unrooted phylogenetic tree of Ribocin homologs. Each node indicates the name of a bacterial taxon carrying an RbcN homolog. Arrow indicates P. aeruginosa Ribocin. The data underlying this panel can be found in S2 Data. (C) AlphaFold-predicted structure of RbcN (PA14_21120), colored by pLDDT score. The unstructured signal peptide is colored yellow. (D) Location in the AlphaFold RbcN structure of four conserved amino acids. H60 and K75 in red, T159 in green, and Y161 in blue color. (TIF) [file pbio.3003790.s001.tif]

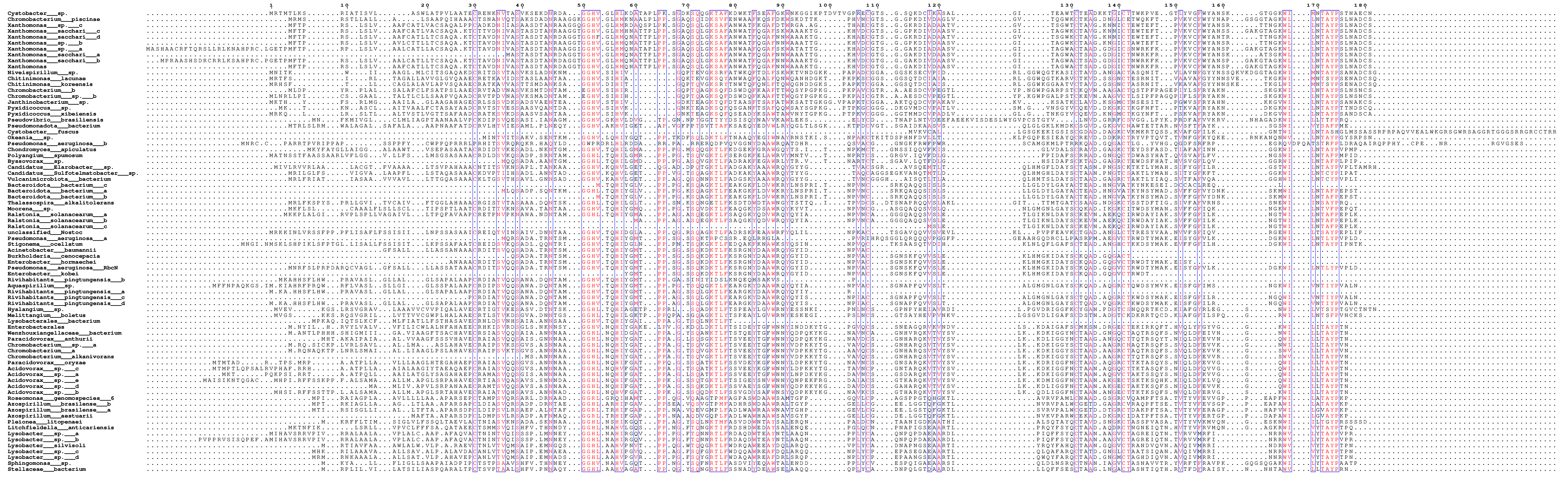

Supplement: S2 Fig — Protein sequence alignment of P. aeruginosa RbcN and the sequence homologs shown in S1B Fig. Identical or similar amino acids are shown in red font and boxed in blue. (TIF) [file pbio.3003790.s002.tif]

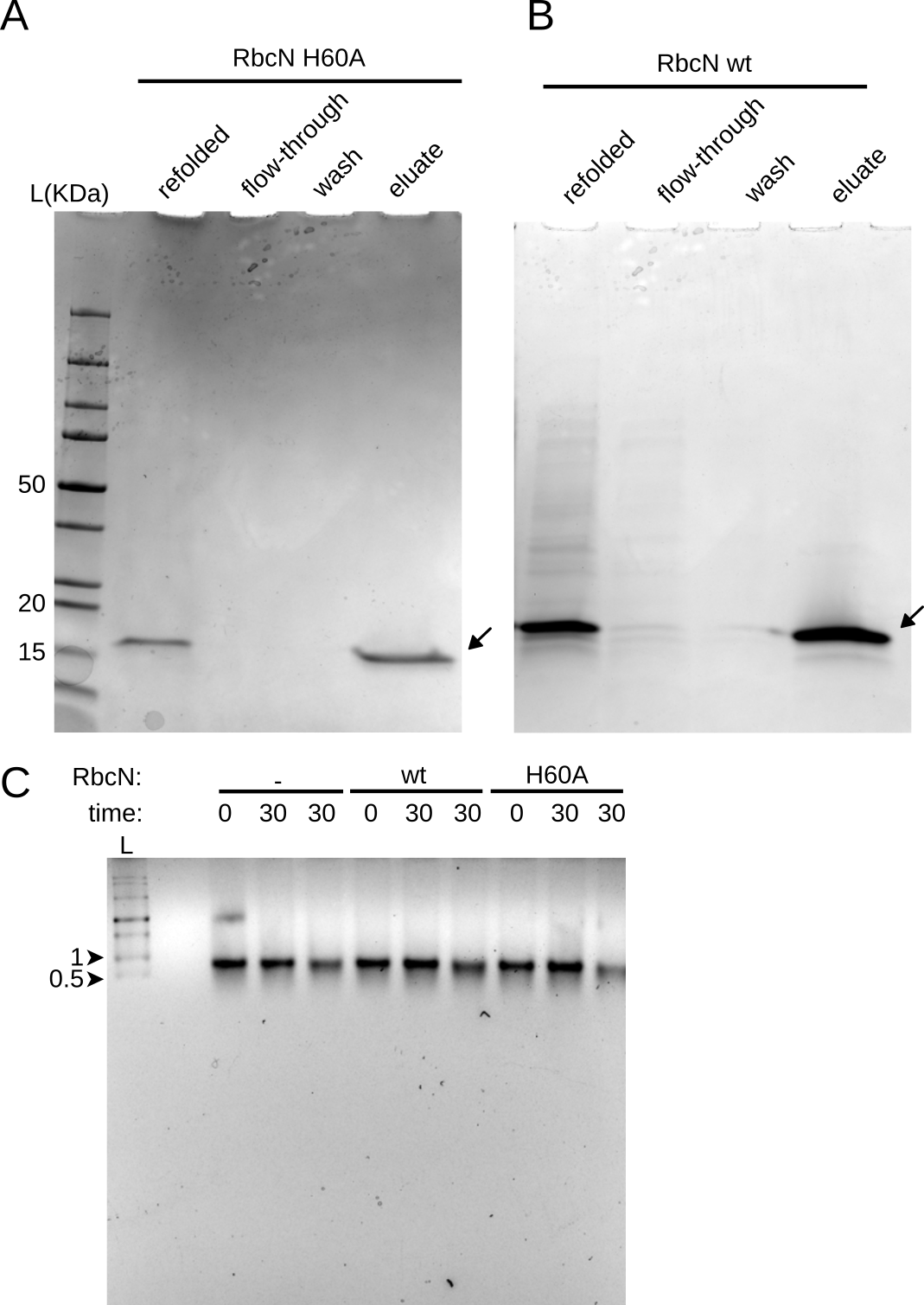

Supplement: S3 Fig — (A, B) Coomassie-stained gels of the recombinant RbcN isolation procedure for wild-type (wt) (A) and H60A mutant (B) protein. Following refolding, RbcN-containing samples were passed through a Ni-NTA column. The protein profiles of flow-through, wash, and eluate samples are shown. The full-length RbcN protein is indicated by arrows. L, protein ladder. kDa, kilodalton. (C) Denaturing agarose gel for an mRNA assayed for cleavage by recombinant RbcN (wt or H60A). Arrowheads indicate RNA ladder markers (× 103 nucleotides). (TIF) [file pbio.3003790.s003.tif]

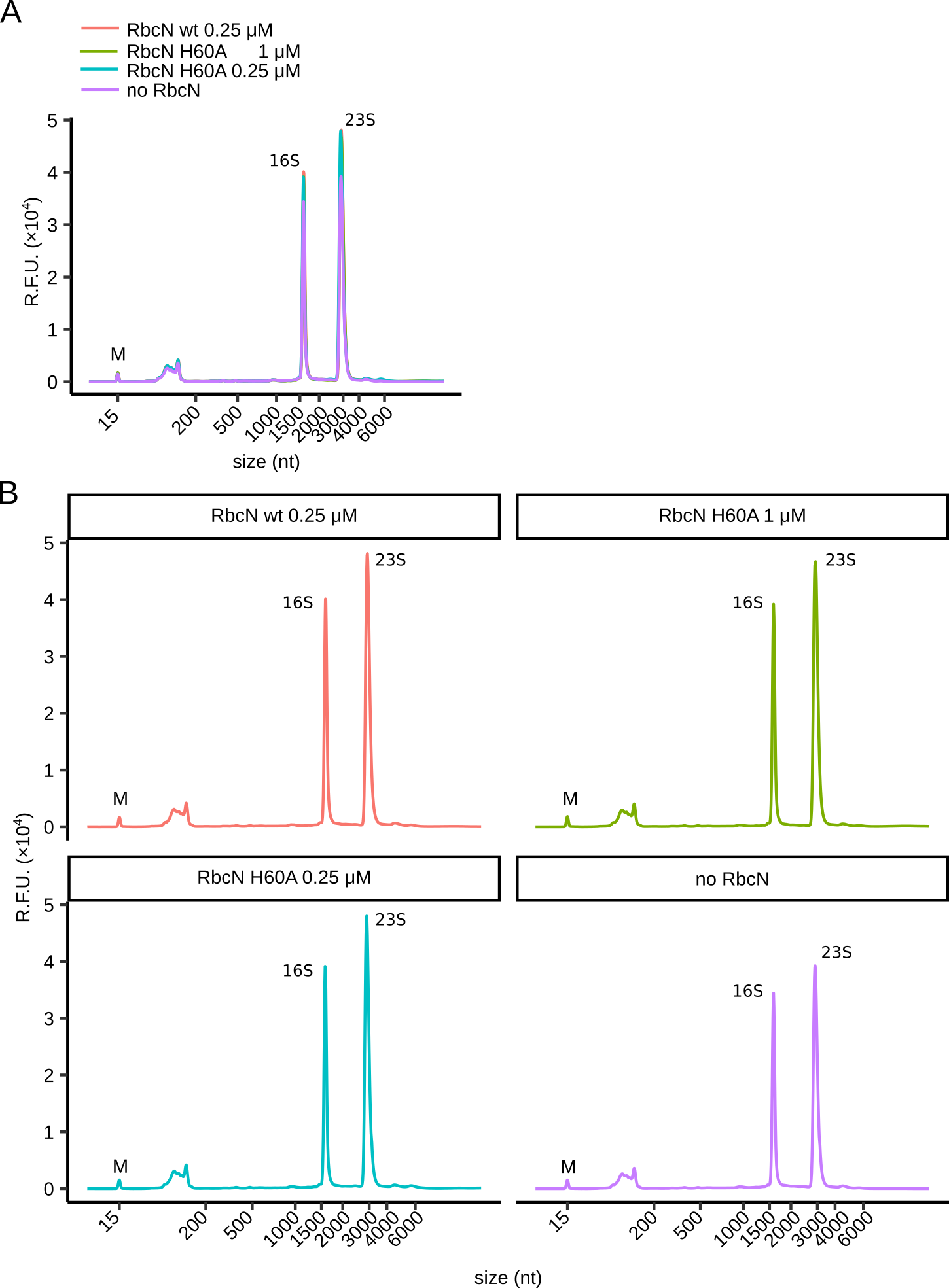

Supplement: S4 Fig — (A, B) RNA profiles of Escherichia coli ribosomes in translationally-competent 30S lysate after treatment with recombinant RbcN wild-type (wt) or H60A mutant RbcN at the indicated concentrations. The individual RNA profiles are overlayed in (A). “M” indicates a 15-nucleotide (nt) marker. R.F.U. represents relative fluorescence units. (TIF) [file pbio.3003790.s004.tif]

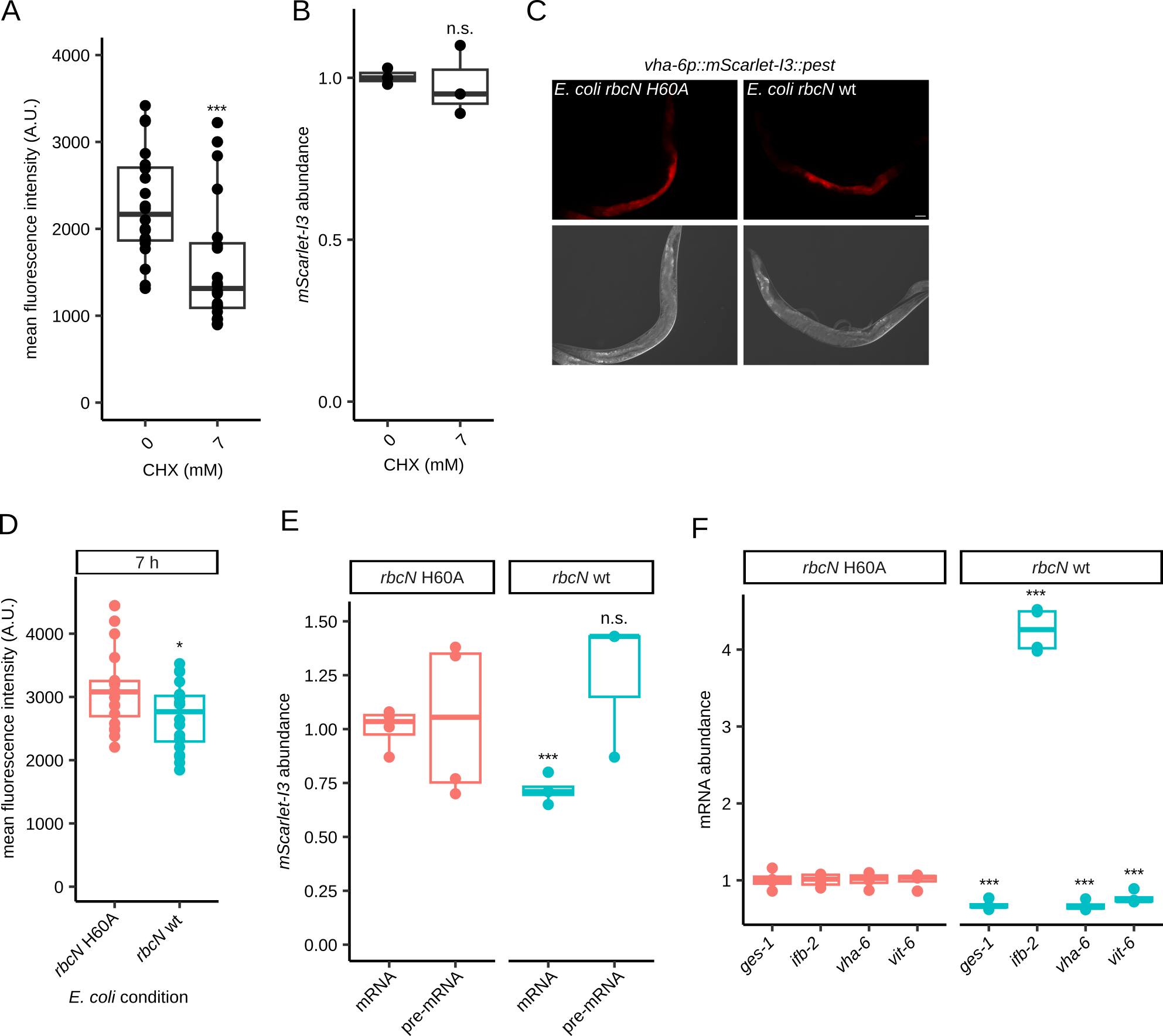

Supplement: S5 Fig — (A) Quantification of the mScarlet-I3::PEST fluorescence upon worm exposure to cycloheximide (CHX) for 3.5 hours (h). (B) Relative abundance of the mScarlet-I3::pest mRNA by RT-qPCR for the conditions shown in (A). (C) Fluorescence microscopy images of mScarlet-I3::pest worms (strain VT4414) exposed to E. coli expressing rbcN wild-type (wt) or H60A for 3 hours. Scale bar measures 50 μm for all images. (D) Quantification of the mScarlet-I3::PEST mean fluorescence intensity for worms exposed to E. coli expressing rbcN wt for 7 or 12 h. (E) Relative abundance of the mScarlet-I3::pest mRNA and pre-mRNA measured by RT-qPCR for the 7 h condition shown in (D). (F) Relative mRNA abundance of four intestine-specific genes (ges-1, ifb-2, vha-6, vit-6) by RT-qPCR for the 7 h conditions shown in (D). The intermediate filament ifb-2 is induced by host responses. The data underlying this figure can be found in S1 Data. “n.s.” denotes not significantly different. “***” indicates a p-value < 0.001 for two-sided Welch t test comparisons. (TIF) [file pbio.3003790.s005.tif]

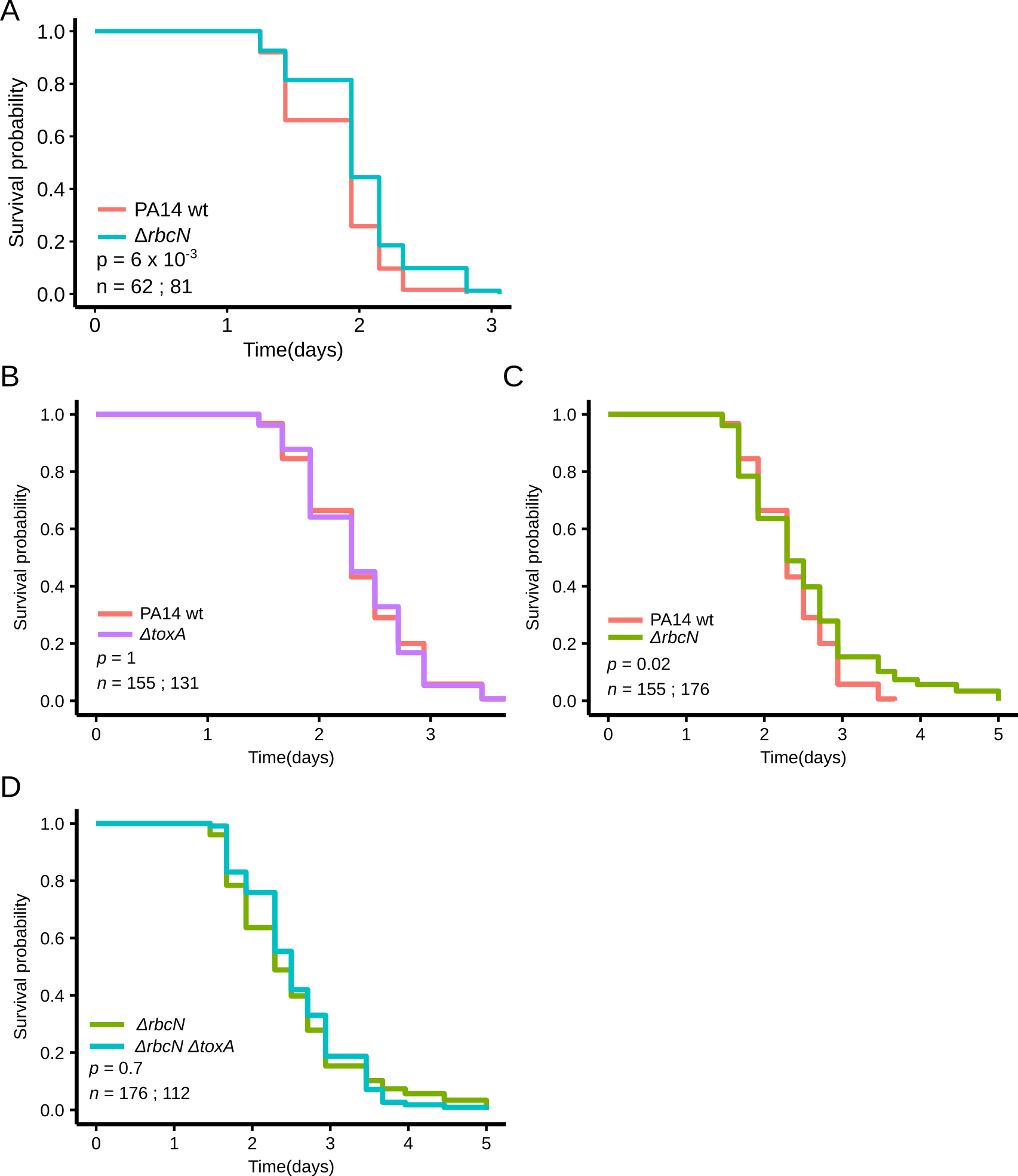

Supplement: S6 Fig — (A–D) Survival curves of adult worms exposed to PA14 (wt, in red) or ΔrbcN. (A,C); ΔtoxA (B); or ΔrbcN ΔtoxA (D). The p-value from a log-rank test curve comparison and n values (individual animals) are shown. The data underlying this figure can be found in S1 Data. (TIF) [file pbio.3003790.s006.tif]

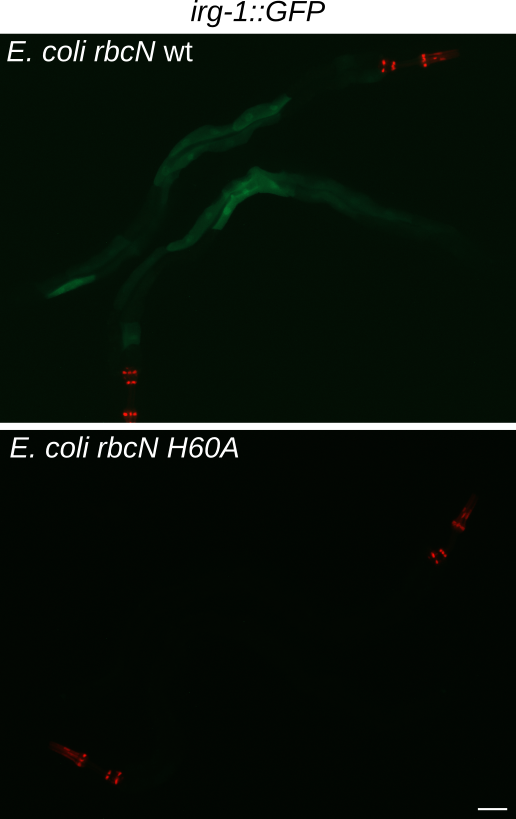

Supplement: S7 Fig — Fluorescence microscopy images of irg-1::GFP worms exposed to E. coli heterologously expressing the rbcN gene, either wild-type (wt) or H60A mutant. Scale bar measures 50 μm for all images. (TIF) [file pbio.3003790.s007.tif]

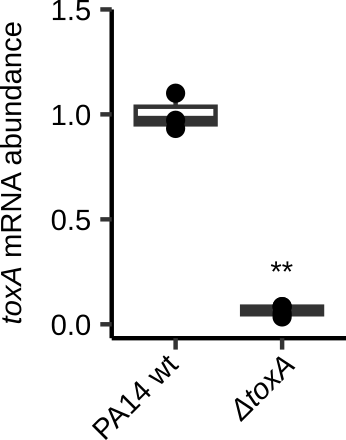

Supplement: S8 Fig — RT-qPCR of relative toxA mRNA abundance in the ΔtoxA strain and PA14 wt. “**” indicates a p-value < 0.01 for a two-sided Welch t test comparison. The data underlying this figure can be found in S1 Data. (TIF) [file pbio.3003790.s008.tif]

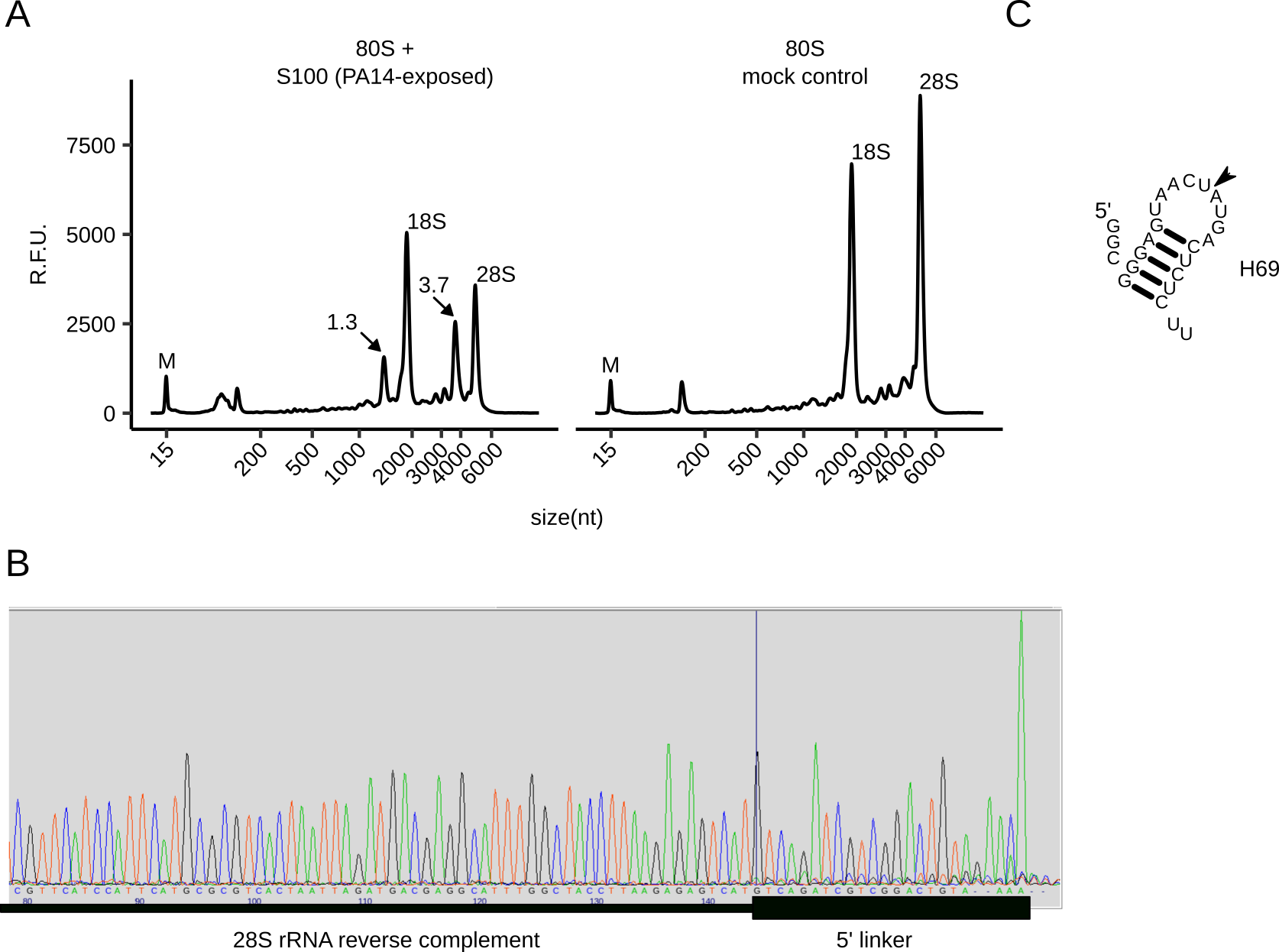

Supplement: S9 Fig — (A) Total RNA profiles of 80S rabbit ribosomes treated with S100 lysate from PA14-exposed worms or mock-treated. Arrows indicate the H69-cleaved fragments with their estimated nucleotide size (×103). (B) Sanger sequencing trace indicating the cloned cleavage site (vertical line) from rRNA of S100-treated rabbit ribosomes shown in (A). (C) Cleavage site (arrowhead) indicated in the Helix 69 rRNA secondary structure. “M” denotes a 15-nt marker. R.F.U. stands for relative fluorescence units; nt, nucleotide; H69, helix 69. (TIF) [file pbio.3003790.s009.tif]
