# Supplementary material for: The Pseudomonas aeruginosa ribonuclease Ribocin cleaves eukaryotic ribosomes at helix 69 to inhibit host translation
Source: PLoS Biol. 2026 May 20;24(5):e3003790. doi: 10.1371/journal.pbio.3003790 (PMC13189344; doi:10.1371/journal.pbio.3003790)
Supplement: S1 Raw Images — Reference to main figure panels is made in the figure. Please refer to the respective legend. (PDF) [file pbio.3003790.s019.pdf]

Raw images for panel 2F

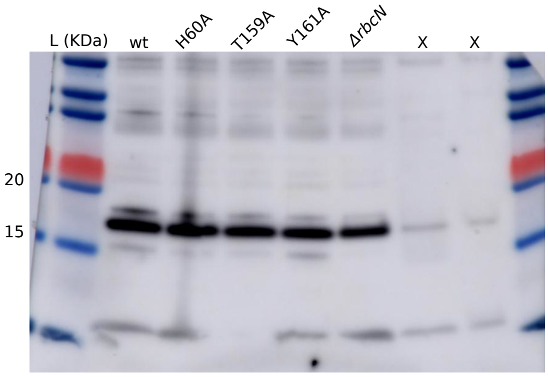

Western blot with α-RbcN

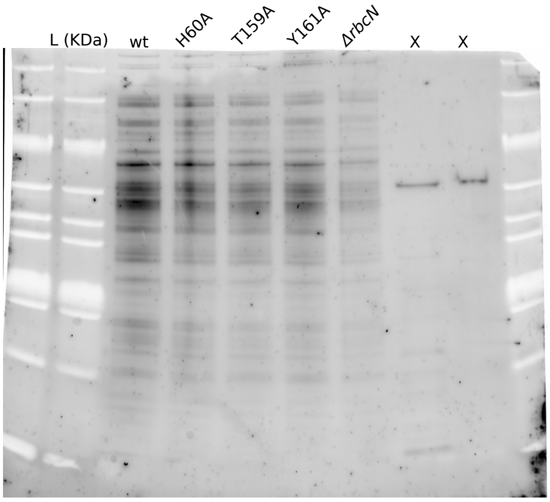

Total protein staining

Raw images for panel S3A

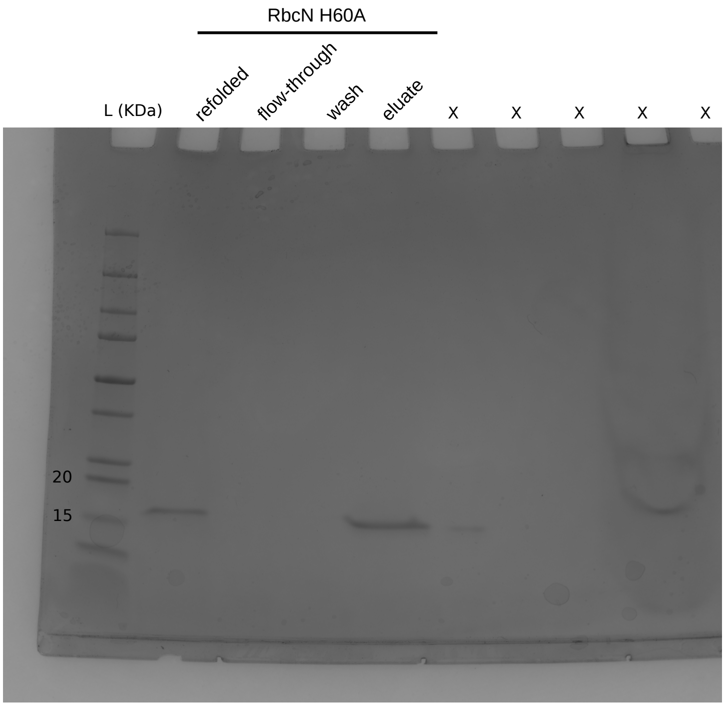

Coomassie-stained gel

Raw images for panel S3B

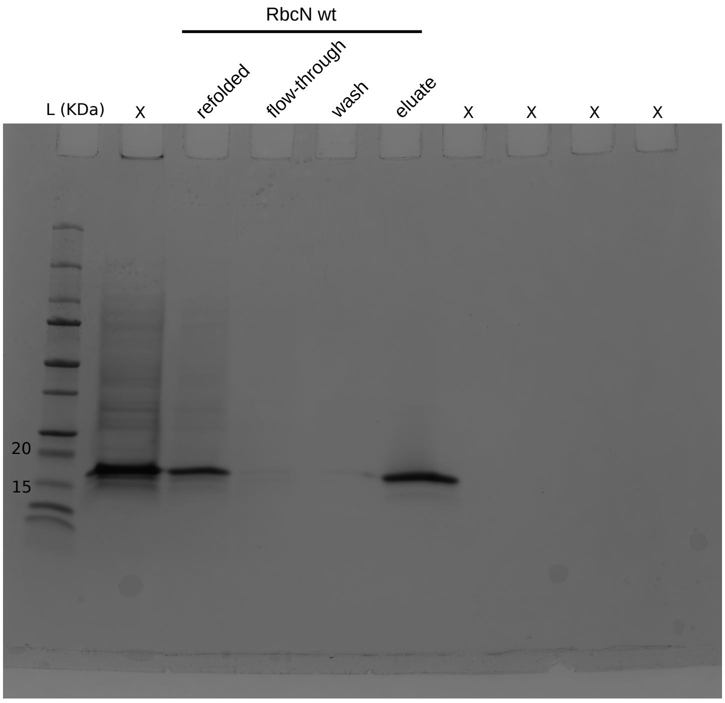

Coomassie-stained gel

Raw images for panel S3C

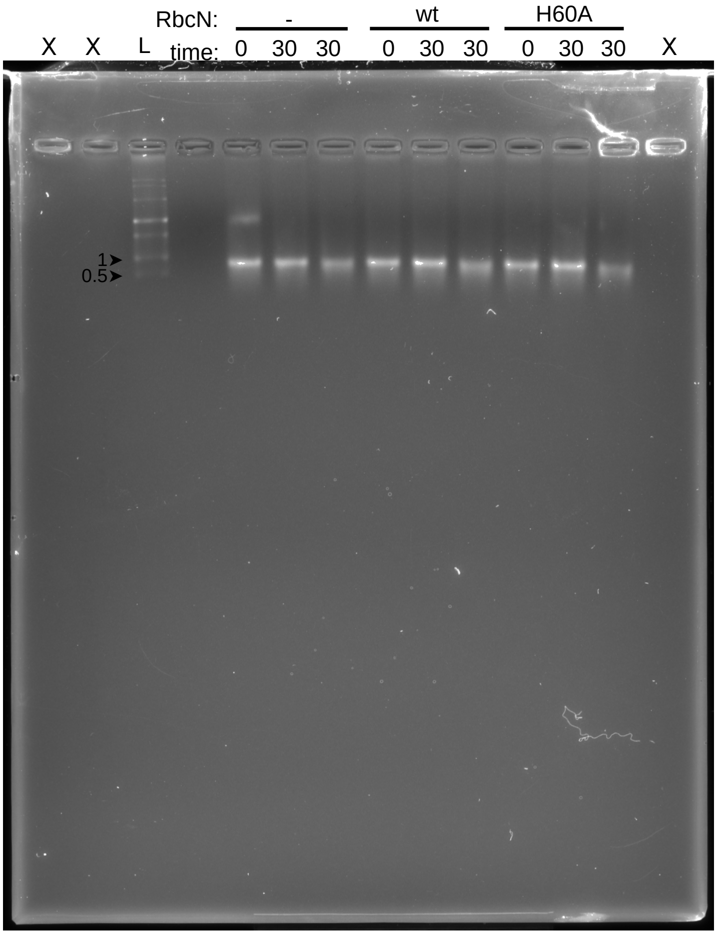

Denaturing agarose gel
