# Supplementary material for: The Pseudomonas aeruginosa ribonuclease Ribocin cleaves eukaryotic ribosomes at helix 69 to inhibit host translation
Source: PLoS Biol. 2026 May 20;24(5):e3003790. doi: 10.1371/journal.pbio.3003790 (PMC13189344; doi:10.1371/journal.pbio.3003790)
Supplement: Attachment — Submitted filename: response_to_reviewers_04_05_2026.docx [file pbio.3003790.s022.docx]

**We thank the referees for their valuable comments and suggestions, which have helped us to improve the manuscript. We have revised the manuscript and figures according to reviewers' suggestions. Our detailed responses are provided below.**

Reviewer #1:

In the manuscript, "P. aeruginosa Ribocin cleaves ribosomal Helix 69 to inhibit host translation", the authors find and characterize a new ribonuclease, Ribocin, showing that it is responsible for the helix 69 cleavage caused by P. aeruginosa that they previously discovered. The study begins by screening infected worm lysates for the H69 cleavage activity using a biochemical approach. Based on the list of candidate proteins, they further screened publicly available C. elegans and P. aeruginosa mutants and found one PA14 mutant in which activity was completely lost. The mutation was in an uncharacterized gene, but with the help of AlphaFold, it was found to resemble proteins in the RNase A superfamily. They then created point mutants in conserved residues crucial for RNase activity and demonstrated a loss of activity in both in vivo (C. elegans lysates) and in vitro (recombinant protein) settings. They also expressed the protein in heterologous systems (E. coli and eukaryotic cells) and demonstrated that it was sufficient to endow the H69 cleavage activity. They also showed that Ribocin's translation-inhibition activity occurs in the presence of this enzyme by adding it recombinantly to an RRL assay and by using in vivo approaches in both human cells and C. elegans. The robustness of the results is bolstered by the use of two complementary methodologies to assess translational inhibition, along with inactive and mutant enzyme controls. Finally, the authors demonstrated that in the worm infection model, where the activity was initially discovered, the Ribocin gene is essential for the translation-blocking activity and is more important than ToxA, a toxin with similar translation-blocking properties. They also observed that loss of the gene results in slightly less PA14 killing of C. elegans. Overall, I find the characterization of a new prokaryotic ribonuclease, with a novel mechanism of acting on eukaryotic cells, to be a fascinating and important contribution. The study is thorough and convincing, with results supported by multiple approaches and rigorously conducted. My only major concern was with how the survival assays were carried out, and is described below.

**Response to Reviewer #1**

**We thank the reviewer for the thoughtful and positive assessment of our manuscript and for recognizing the significance and rigor of our study.**

Major Comments
1) Survival Assays: Due to the inherent variability associated with these assays, it is common practice in the field to include multiple biological replicates, typically three. This was not done in this study. Rather, single assays are shown with a large n (150-200). The large sample size was probably necessary to detect the very small effect of the loss of the rbcN gene on survival. However, I think it is really important to have multiple biological replicates to further probe the influence of this one gene on survival and to avoid overinterpretation. It will not, in my opinion, detract from the impact of this manuscript if the effect proves inconsistent across multiple replicates. Pseudomonas possesses numerous virulence factors, and it is logical that losing just one may be insufficient to affect host survival, a complex, multifactorial phenotype.

**Response to major point 1:**

**The survival assays presented in manuscript were conducted with at least three independent biological replicates and the corresponding results are representative, i.e., consistent across all the independent biological replicate experiments. Two biological replicates of the survival curve for *ΔrbcN* are shown in the manuscript sent to the reviewers (Fig 5A and Fig S6B). We have now included in the manuscript a third biological replicate of the *ΔrbcN* survival curve (see updated Fig S6A). In all the three biological replicates, a small but statistically significant effect of *ΔrbcN* on worm survival was detected.**

**In the revised manuscript, we now explicitly state that we carried out biological replicates for all survival analyses. We have modified the ‘Worm survival analysis’ section of ‘Materials and Methods’ to include the statement (lines 699-700 in the revised manuscript): “The survival assays were performed with three or more independent biological replicates, and the results were consistent across all independent replicate experiments.”**

Minor Comments
1) Figures 4I and J would be further clarified by listing the time point in the panels, so that the reader does not need to refer to the legend to understand their difference. In fact, they could be combined into a single panel with both time points displayed together.

**Response to minor comment 1: the time points are now shown in the updated panels 4I and 4J to facilitate reading without referral to the legend. These two panels correspond to experiments that were not conducted side-by-side and therefore we prefer to present them in separate panels.**

2) Figure 4 shows all the conditions using both the mScarlet reporter strain and the puromycin assay, except for the PA14 mutants shown in Figures 4I and 4J. Was there a reason the mScarlet assay was not additionally used in this experiment? If the authors have it, could it be included here or put in the supplement?

**Response to minor comment 2: We established that the mScarlet-I3 and puromycin assays led to consistent results, as shown in panels 4G and 4H, where worms were exposed to 3 bacterial strains (PA14 wild type, *ΔrbcN* and *Δ3QS*) for 12 h. However, the puromycin assay is a more informative readout of ongoing global translation because Puromycin-labelled nascent peptides are measured, as opposed to a gene-specific readout like the mScarlet-I3 assay. Moreover, the Puromycin assay does not depend on protein folding and maturation, unlike the mScarlet-I3 assay, so it is better suited for characterizing overall translation upon short exposures of worms to bacteria,. For these reasons, we only used the puromycin assay for the experiments of panels 4I and 4J, which also include a shorter exposure of 5 h to bacteria (panel 4I). We now emphasize the “global translation” readout point in the Results section.**

----------------------------------------------------------------


Reviewer #2:

In this work, Ambros and coworkers identify and characterize Ribocin/RbcN, a nuclease of P. aeruginosa that specifically cleaves H69 of eukaryotic ribosomes. They show that RbcN is distally related to CdiA (another bacterial endonuclease toxin) and is sufficient for H69 cleavage and translation inhibition in C. elegans. They purified the protein and showed that it inhibits translation in vitro (rabbit reticulysate), with an IC50 value of 33 nM. They also provide evidence that RbcN inhibits translation in human and worm cells. In the worm, this activity is revealed in the absence of ToxA, at early (5 h) exposure time. Finally, the authors show that RbcN contributes to P. aeruginosa virulence in the worm and is critical for host IRG-1 response pathway. Overall, this is nice work, and I have only a few recommendations for the paper.

**Response to Reviewer #2**

**We thank the reviewer for its positive assessment of the manuscript and for the recommendations.**

Specific comments:
RbcN cleaves H69 of various eukaryotic ribosomes. Does it act on extracted rRNA? Unmodified (transcript) rRNA? Does RbcN also target H69 of archaea and/or bacteria? Some further discussion of the enzyme's specificity would be appropriate, even if the questions remain open.

**Response to specific comment: At the request of the reviewers, we have investigated Ribocin activity on bacterial ribosomes. We have found that Ribocin does not detectably cleave *E. coli* 70S in translation-competent S30 extracts. The result is now shown in the revised figure S4, with a discussion of this result in the ‘Results’ section (lines 208-210 in the revised manuscript) and ‘Discussion’ section (lines 363-366). The likely reason for the lack of activity is in the structural differences between bacterial and eukaryotic ribosomes; despite the high conservation of the essential decoding center nucleotides, neighboring nucleotides and ribosomal protein loops and tails (e.g., uS12 and uS19) differ among bacteria and eukaryotes. A detailed structural and mechanistic comparison is, however, beyond the scope of this work.**

**In the present manuscript and in the previous study (Vasquez-Rifo, et al., 2020, PLoS Biology), we settled on using the ribosome, the natural substrate of Ribocin, for all our experiments. We previously tested and observed no Ribocin-mediated H69 cleavage using naked H69 RNA hairpins (unpublished observations). In ongoing studies, we are establishing the molecular mechanisms that confer specificity to the ribosomes as the substrate of Ribocin-mediated H69 cleavage.**

Fig. 5A shows the survival rates of worms exposed to PA14 and the del-rbcN strain. Missing is an appropriate negative control (i.e., avirulent P. aeruginosa or E. coli).
**Response to specific comment: In the context of the survival rate of worms exposed to *ΔrbcN* or wild-type PA14, a “negative control” should address the possibility that the observed survival effect of *ΔrbcN* arises not from the deletion of *rbcN* itself, but from other factors, such as unintended background mutations introduced during genome editing. Comparisons of survival curves between PA14 wild type and *ΔrbcN* with avirulent strains (e.g., *E. coli*) would not inform whether such potential confounding factors are present in our experiments.**

**By contrast, multiple lines of evidence in our study argue against the possibility that the *ΔrbcN*-associated survival phenotype is unrelated to the deletion of *rbcN*. First, we performed whole-genome sequencing on all genome-edited bacterial strains (including *ΔrbcN*) and detected no unintended background mutations introduced during genome editing (Materials and Methods, lines 436–438). Second, the *ΔrbcN* deletion was generated independently in two strains—*ΔrbcN* and *ΔrbcN ΔtoxA*—and both exhibited the same phenotype in worm survival assays (Figure S6D). We now explicitly state this in the Materials and Methods section (“Bacterial strains and genetics,” lines 440–441): “The *ΔrbcN* and *ΔrbcN ΔtoxA* strains were generated independently from the parental PA14 wild type.” Third, the *ΔtoxA* strain, generated using the same methodology as *ΔrbcN*, showed no effect on worm survival, indicating that the genome-editing procedure itself does not introduce detectable changes in bacterial virulence. Finally, all survival curves for PA14 wild type (Fig. 5A; Fig. S6A–B) show a median worm survival of approximately 2 days, consistent with the expected outcome under SK assay conditions.**

**Together, these observations support the conclusion that the worm survival phenotype associated with *ΔrbcN* is specifically attributable to the deletion of *rbcN* and not to unintended background effects.**

In Fig. 4I-J, the data are "tubulin-normalized," based on the legend. I presume this means that the data of I and J are directly comparable, despite that the Y units are arbitrary. If so, there is a dramatic drop in puro-peptide signal across the board, for even the avirulent strain. Why would this be? Perhaps I am missing something, but regardless, this section of the manuscript should be clarified.

**Response to specific comment: The data for 4I and 4J are not directly comparable as they represent different experiments with separate gels and membranes. Therefore, variations in the western blot procedures are expected to result in differences in the values for the westerm blot signals – both for anti-puromycin antibody and for anti-tubulin. Furthermore, as noted by the reviewer, the blot signals of worm samples analyzed after 12 h are lower than those of worms analyzed after 5 h. It is plausible that there is a drop in the measured translation between 0 and 12 hours, even for the the avirulent *Δ3QS* strain, because in addition to the Western results, we have also noticed decreased mScarlet-I3 fluorescence along those times (unpublished observation). Nevertheless, the aim of the experiment was to compare the effects among bacterial strains at the selected time points, so we did not aim to compare signals from different western blots, and we did not optimize experimental conditions to achieve that goal. For example: 1) we did not use the exact same number of worms for different westerns (instead, the same amount of worms was used for each lane within each western); 2) HRP reaction exposure settings were not fixed, but determined automatically by the digital imager; 3) we did not use internal standards to facilitate comparison between separate westerns.**

**As the western blot signals of panel 4I were comparatively low, we have repeated the experiment shown in panel 4I. The new western blot (updated panel 4I) reproduced the relative results from the previous blot, yet yielding higher signal values that are closer to those in panel 4H (both panels are from worms exposed for 12 h to *P. aeruginosa* PA14).**

Fig. 3B, right panel. The three traces seem intentionally offset via total signal, to see the three colors. This is a bit confusing, especially when different concentrations (albeit of protein) are listed in the key. I would suggest that all four traces (including wt, left panel) be normalized to total signal and then positionally offset (vertically), so the effects are clearer.
**Response to specific comment:**

**The electrophoretic traces in panel 3B were not offset, but the sample ‘RbcN H60A 0.25 microM’ (green line) had less recovered RNA content compared to the other traces, likely leading to the appearance of an offset trace. We have repeated the experiment to fix this potential confusion with more homogeneous trace data. The new data (see updated panel 3B) sustains the same results as before, and the traces are represented as an overlay (no offset) of the four conditions tested (updated panel 3B). As noted in the updated figure legend, the electrophoretic traces for the control and RbcN H60A conditions are largely overlapping.**

Check callouts to Fig. 3B-C (page 9, second paragraph). It seems like panel B, right and left, is what is meant?
**Response to minor comment: We have corrected the Figure 3 legend. There is now only one panel shown in Fig 3B and hence no mention of ‘right and left’ is required. The updated legend now includes a mention of ‘right and left’ for panel 3C.**

Typo: Fig. 2 legend, "starts point to unspecified bands."
**Response to minor comment: We have corrected the Figure 2 legend which now reads “... stars point to non-specific bands."**
----------------------------------------------------------------


Reviewer #3:

The manuscript by Vasquez-Rifo et al. describes the characterization of a Pseudomonas aeruginosa ribonuclease, Ribocin, that carries out the cleavage of the large ribosomal RNA of the C. elegans host. This is an important and rigorous follow-up study to the authors' previous observation that the pathogen P. aeruginosa can inhibit C. elegans host translation by cleavage of the large ribosomal RNA at helix 69. The study is logically presented and experiments establish that Ribocin is necessary and sufficient even when heterologously expressed in E. coli, to carry out the translational inhibition by cleaving the large ribosomal RNA of C. elegans. I only have one comment that may help the audience better appreciate the physiology of the observed interaction—can the authors discuss further whether Ribocin is secreted by bacteria, and if so, how might it be taken up by the intestinal cells of C. elegans, as P. aeruginosa itself is thought to remain largely in the intestinal lumen during infection.

**Response to Reviewer #3**

**We thank the reviewer for the positive evaluation of our manuscript and for the suggestion.**

**In the updated manuscript, we have added a discussion of the putative mechanisms of Ribocin delivery to the worm's cytoplasm (lines 368-374), and cite a new reference [43].**

----------------------------------------------------------------

Reviewer #4:

In this manuscript, Vasquez-Rifo et al. carried out comprehensive characterization of P. aeruginosa H69 nuclease, which is named Ribocin. Specifically, the authors identified Ribocin in vivo and performed ribosomal RNA cleavages both in vivo and in vitro, demonstrating that ribosomes from various eukaryotic organisms are the effective substrates of Ribocin. In addition, the authors demonstrated that Ribocin inhibits eukaryotic protein translation in vivo, and it contributes to the virulence of the encoded bacteria. The experimental data provided are abundant and of high quality. This reviewer particularly appreciated the fact that, in addition to ribotoxins targeting sarcin-ricin loop (SRL) and the decoding center of the ribosome, a new ribotoxin specifically targeting H69 has been discovered and comprehensive characterized. Therefore, the study described in the manuscript is highly significant.

This reviewer has no major issues with the manuscript. However, the manuscript could be improved if the authors can address the following two minor issues. Those two issues are to address ribosomal damage in a broader context, e.g., both eukaryotic and bacterial ribosomes.

**Response to Reviewer #4**

**We sincerely thank the reviewer for the thoughtful and highly positive evaluation of our work and for the two constructive suggestions.**

1. Line 49: After the discussion of ribotoxins targeting SRL and before small molecule toxins inhibiting protein translation, the authors should add brief discussion of ribotoxins that target the decoding center. **Response to minor comment: We now mention the colicin E3 and CdiA-CTECL nucleases—that target the decoding center of bacterial ribosomes—in the manuscript’s introduction (lines 49-51 in the revised manuscript). Three new literature citations are included with the above-mentioned manuscript modification (references [5-7]).**

**We also discuss the structures of other bacterial-ribosome-targeting nucleases in the Discussion section (lines 363-366), with six new literature citations (references [5] and [47-51]).**

2. Discussion section: It might be helpful to have a brief discussion why Ribocin does not cleave bacterial ribosome at H69. Sarcin is also eukaryotic ribosome specific. But Sarcin can also cleave bacterial ribosome, although the activity is significantly lower. Is there any data showing that Ribocin does not cleave bacterial ribosome at all?

**Response to minor comment: At the request of the reviewers, we have investigated Ribocin activity on bacterial ribosomes. We have found that Ribocin does not detectably cleave E. coli 70S in translation-competent S30 extracts. The result is now shown in the revised figure S4, with a discussion of this result in the ‘Results’ section (lines 208-210) and ‘Discussion’ section (lines 363-366). The likely reason for the lack of activity is in the structural differences between bacterial and eukaryotic ribosomes; despite the high conservation of the essential decoding center nucleotides, neighboring nucleotides and ribosomal protein loops and tails (e.g., uS12 and uS19) differ among bacteria and eukaryotes. A detailed structural and mechanistic comparison is, however, beyond the scope of this work.**
